# Quality of life assessment in domestic dogs: An evidence-based rapid review

**DOI:** 10.1016/j.tvjl.2015.07.016

**Published:** 2015-11

**Authors:** Z. Belshaw, L. Asher, N.D. Harvey, R.S. Dean

**Affiliations:** Centre for Evidence-based Veterinary Medicine, School of Veterinary Medicine and Science, University of Nottingham, Sutton Bonington Campus, Loughborough, Leicestershire, LE12 5RD UK

**Keywords:** Dog, Quality of life, Validation, Review, Welfare

## Abstract

Assessment of quality of life (QoL) is an important, increasingly popular outcome measure in veterinary research and practice, particularly in dogs. In humans, QoL is commonly assessed by self-reporting and since this is not possible for animals, it is crucial that instruments designed to measure QoL are tested for reliability and validity. Using a systematic, replicable literature search strategy, the aim of this study was to find published, peer-reviewed instruments for QoL assessment in dogs and to assess the quality of these. CAB Abstracts and PubMed were searched in July 2013 using terms relevant to dogs, wellbeing and QoL. Inclusion and exclusion criteria were applied. When instruments were not published in full, authors were contacted to obtain them. Criteria were applied to assess the quality, validity and reliability of the 52 instruments obtained. Twenty-seven additional instruments used in peer-reviewed publications were not included because they had not been fully described in the publication or were not provided by authors upon request.

Most of the instruments reviewed (48/52) were disease-specific rather than generic. Only four publications provided a definition of QoL or wellbeing. Only 11/52 instruments demonstrated evidence of assessing reliability or validity, and the quality of these instruments was variable. Many novel, unvalidated instruments have been generated and applied as clinical outcomes before it was known whether they measured QoL. This rapid review can be used to identify currently available and validated canine QoL instruments, and to assess the validity and quality of new or existing instruments.

## Highlights

• We provide a clear methodology for the assessment of validity, reliability and quality of life (QoL) instruments.
• Few authors of publications containing a QoL instrument define quality of life.
• Few of the instruments published for the assessment of QoL in dogs are validated.
• The quality of validated instruments for the assessment of QoL in dogs is variable.

## Introduction

Assessing the quality of life (QoL) of companion animals is a ‘central part of veterinary practice’ (Yeates and Main, 2009). The success of an intervention or treatment can be defined by the owners' perception of their pet's subsequent improvement in QoL (Levine et al., 2008). Poor QoL as perceived by owners has been reported as a common reason for euthanasia of British pets (Edney, 1998). An assessment of QoL is likely to provide information to owners and veterinarians which complements traditional measures of intervention success, such as median survival times (Spofford et al., 2013). Veterinary QoL instruments have been created for several species including cats (Niessen et al., 2010) and pigs (Wiseman-Orr et al., 2011), but veterinary QoL instruments have most commonly been developed for use in dogs.

Measuring QoL in animals can be challenging and is hampered by the current lack of a suitable definition of QoL in animals. The widely accepted definition of human QoL proposed by the World Health Organization^1^ is not appropriate for use in domestic species as it includes references to culture and values. Since consensus does not exist, any publication describing a measurement of QoL for use in animals should provide a definition; yet the term is infrequently defined in existing publications (McMillan, 2000). Additionally, there can be confusion between broader QoL and health related quality of life (HRQoL), with the latter referring to ‘*the specific effect of a medical condition on an individual's health*’ (Cella, 1992). A QoL assessment should encourage consideration of all aspects of a pet's life, not just its physical health (McMillan, 2000).

The QoL of animals can only be assessed using proxy reports or direct observations (McMillan, 2000). In small animal veterinary QoL assessments, owners frequently act as proxies (Taylor and Mills, 2007). In this role, owners recognise and interpret their animal's behaviour – an assessment that is likely to be subjective (for review, see Yeates and Main, 2009). For human patients where self-reporting of QoL is not possible, an adult who knows the individual well acts as a proxy (Eiser and Morse, 2001). Studies where self and proxy reports were compared (Janse et al, 2005, April et al, 2006, Vetter et al, 2012) found that proxy reporters might rate non-physical aspects of QoL lower than ratings by the self-reporter; this is known as the ‘disability paradox’ (Ubel et al, 2005, Schwartz et al, 2007). It is unclear whether the ‘disability paradox’ also exists in veterinary medicine. While in human QoL instruments, a proxy can reliably assess physical aspects, this area has been largely unexplored in veterinary medicine. Recent publications have reported discrepancies between canine lameness, as reported by owners and veterinarians, and an objective measure (Innes, Barr, 1998, Conzemius, Evans, 2012, Brown et al, 2013b).

Five steps have been identified in the development and application of a QoL assessment instrument (Yeates and Main, 2009), and a sixth step of rigorous assessment of validation and reliability has been recently proposed (Spofford et al., 2013). If an instrument is not validated, we cannot be certain that is truly measuring what it is designed to measure. Previous reviews have also commented on the need for validation of veterinary QoL instruments (McMillan, 2000, Wojciechowska, Hewson, 2005, Hewson et al, 2007, Scott et al, 2007, Yeates, Main, 2009, Giuffrida, Kerrigan, 2014). Instrument validation is a rapidly evolving field that includes complex definitions; the types of validation that can be performed depend on the design of the instrument. Even a well-validated instrument might still be poorly designed, so it is important to evaluate the quality of the instrument as well as its validation (Gill, Feinstein, 1994, Guyatt et al, 1997, Locker, Allen, 2007). Bias can occur throughout the process of assessment, and potential sources should be recognised and acknowledged (for a review of sources of bias, see Choi and Pak, 2005^2^).

Evidence synthesis, combining multiple sources, can be the most reliable type of evidence in evidence-based medicine (Centre for Reviews and Dissemination, University of York, 2009) and systematic reviews are becoming more frequent in veterinary medicine.^3^ A rapid review differs from a systematic review in that it is conducted over a shorter time course and typically covers a more limited range of sources. A rapid review still includes explicit search strategies and methodology as well as rigorous, structured appraisal of the evidence found (Khangura et al., 2012). It is therefore a useful alternative to systematic review, particularly in emergent fields.

To the authors' knowledge, there has been no comprehensive, rigorous and replicable synthesis of peer-reviewed QoL instruments used in dogs. The aim of this rapid review was to find published, peer-reviewed instruments used for QoL assessment in dogs. For each instrument, the validation and assessment of reliability performed in all publications describing its use were appraised using a checklist, and the quality of the instrument was also appraised. The objective was to perform a repeatable, rapid review of all novel, peer-reviewed English language assessment methods for assessing QoL or wellbeing in dogs.

## Materials and methods

### Definition of terms

For the purpose of this review, QoL was operationally defined by the authors as ‘*an individual's satisfaction with its physical and psychological health, its physical and social environment and its ability to interact with that environment*’. In this definition, health was taken to mean ‘*the state of being free from illness or injury*’,^4^ and satisfaction to be ‘*the fulfilment of one's individual needs, or positive mood or valence derived from this*’.^4^

A QoL assessment instrument was defined as: (1) any question, or set of questions, directed to a veterinarian, clinical investigator, owner or caretaker, used by the authors to assess, or comment on, the QoL of dogs; or (2) any other methodology used to gather directly observed data for the same purpose. An item referred to a single question, such as ‘*How is your dog's appetite?*’ and a domain identified a broader area to be measured, such as comfort, which can be measured by accumulating responses from multiple question items. A recall period was the specific time window that a respondent was instructed to reflect upon to answer a question. To aid the readability of this review, the umbrella term ‘QoL’ was used to encompass wellbeing, quality of life and their synonyms. For the purposes of this paper, these terms are described as ‘keywords’ when discussing their use in the abstracts of publications searched.

For an instrument to be defined as validated, at least one aspect of validation must have been intentionally achieved. An unvalidated instrument was defined as one where no evidence of validation was provided. A novel instrument was defined as one that had not been previously published in a peer-reviewed journal.

### Search methods

A search of CAB Abstracts (1910-2013) and PubMed (1948-2013) using the OVID interface was performed in July 2013. The abstract, title, original title, broad terms and heading words were searched using terms relevant to dogs (dog, dogs, canine, canines or canis), wellbeing (wellbeing, well-being, well being) and quality of life (quality of life, QoL, quality-of-life). The searches were linked with Boolean terms as (dog OR dogs OR canine OR canines OR canis) AND (wellbeing OR well-being OR well being OR quality of life OR QoL OR quality-of-life).

### Inclusion criteria

The inclusion criteria for publications were as follows: (1) be in the English language; (2) be in a peer-reviewed journal accessible by the authors; (3) contain one of the keywords in the abstract; (4) contain a form of instrument for the assessment of QoL; (5) be the first published report of that instrument, and (6) be available to the authors in full. Where an instrument had several parts, and only one was novel, only the novel part was reviewed. For publications where the full publication or instrument was not available, a search was conducted online. If the publication or instrument was not found, authors were contacted by email in the following order: first; last; any. Where email addresses were not printed in the relevant publication, they were obtained, where possible, by an Internet search, and authors were given 4 weeks to reply. The publication was excluded if not provided by its authors within this period.

### Exclusion criteria

The exclusion criteria for publications were as follows: (1) not written in English; (2) not published in a peer-reviewed journal; (3) did not contain the ‘keywords’ in the abstract; (4) did not contain an instrument; (5) had been previously published in an earlier publication already found in this search, and (6) unavailable to the authors.

### Application of inclusion and exclusion criteria

A single author (ZB) performed the initial search and applied the inclusion and exclusion criteria to all publications. To ensure consistency, a random sample of 20% of all publications that met the first three inclusion criteria was independently appraised according to the other inclusion/exclusion criteria by a second author (RD). Information on instrument purpose, design and use (Table 1) was extracted by one author (ZB) from all publications that met the inclusion criteria.

### Evaluation of reliability, validity and quality

Each QoL instrument was assessed for reliability and validity. Using the complete manuscripts, the presence or absence of validation and the level of validation (where present) were independently scored by two authors (ZB, NH), using checklists developed for the purpose (Table 2, Table 3), adapted from Taylor and Mills (2006). Each criterion was scored as present, absent, or not applicable. Where ZB and NH were not in agreement on a score, a third author (LA) scored the criterion in question and consensus was reached after discussion.

Evidence of additional or subsequent validation was ascertained by searching Scopus (January 2014) for citations of each of the ‘validated’ publications. The same checklist of reliability and validity was applied to these publications and validation that was scored as present was recorded for each instrument across all its uses. Where there was disagreement between the two scorers, a third scorer (LA) was asked to make the final decision.

The quality of the validated instruments was assessed by one author (ZB) using 10 criteria (Table 4) adapted from those developed for the purpose in human QoL appraisal (Gill, Feinstein, 1994, Guyatt et al, 1997, Locker, Allen, 2007). Each validated instrument, as available for review, was scored against the questions with the following possible results: Yes/No/Not stated/Not applicable/Definition unclear.

## Results

**Fig. 1:**
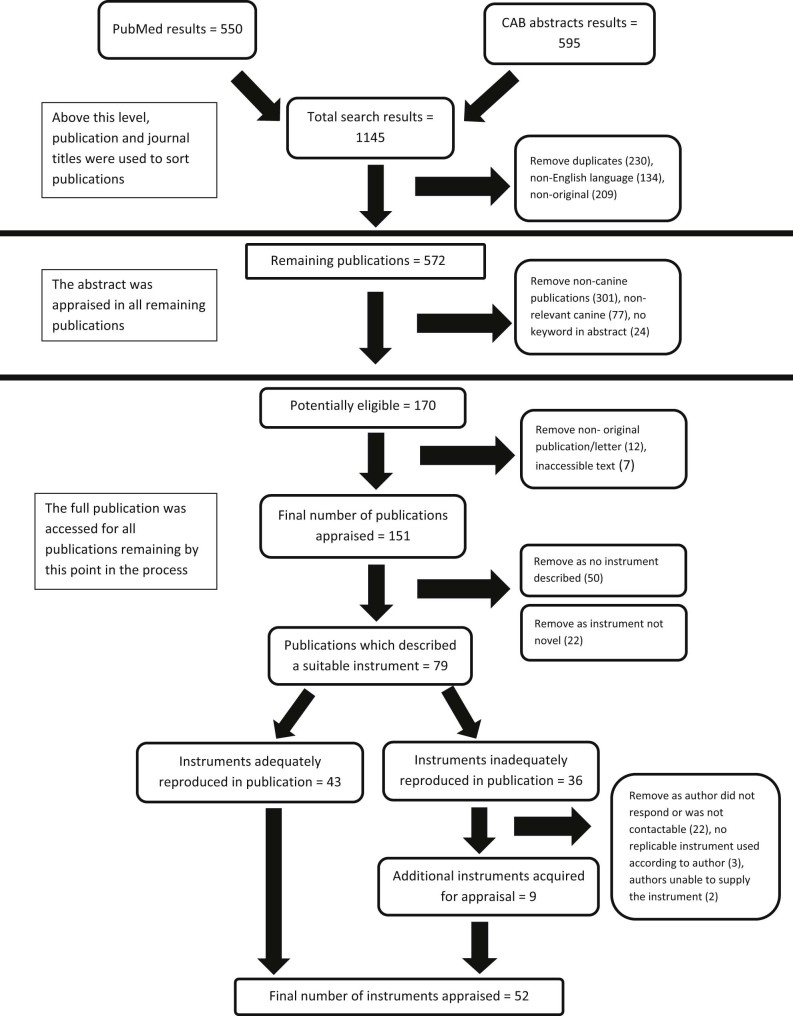
Summary of the systematic application of inclusion and exclusion criteria.

The initial search returned 1145 unique publications, of which 151 met inclusion criteria 1–4 and were assessed at the level of the whole publication (Fig. 1). After systematically excluding publications that did not meet the other inclusion criteria, 52 remained. There was complete agreement between the two reviewers as to which publications met the inclusion criteria. These publications dated from 1987 to 2013, with the majority published since 2003. Publications appeared in 19 unique journals with the highest number of instruments in the *Journal of Small Animal Practice* (*n* = 12), and the *Journal of the American Veterinary Medical Association* (*n* = 10).

### Unvalidated instruments

Of these 52 publications, 41 used instruments to assess QoL without a description of their prior validation (Supplementary Table S1). Thirty-four of the 41 instruments were fully reproduced, adequately described or referenced in that publication. Instruments ranged from a single question such as ‘*What is your pet's quality of life now?*’ (Craven et al., 2004) to long multi-item questionnaires (Lord and Podell, 1999). Fourteen of the 41 instruments were for the assessment of veterinary oncology patients; six were used in each of veterinary cardiology and neurology. Thirty-five of the 41 instruments were for completion by a dog owner, five by a veterinarian, and one by both veterinarian and owner. Ten of the 41 publications contained details on why items were included in the instrument. Craven et al. (2004) was the only publication that defined QoL or wellbeing. Potential sources of bias were seldom acknowledged.

### Validated instruments

Eleven of the 52 publications (21%; Table 5) described the initial process of validating an instrument. One instrument (Schneider et al., 2010) was intended for generic QoL assessment. Eight instruments were validated for use in specific disease types; the other two instruments (Mullan, Main, 2007, Yeates et al, 2011) were designed to raise awareness of welfare considerations. Four of the 11 instruments were fully reproduced, allowing for immediate appraisal and further use. Instruments varied in length from the short instrument of Yeates et al. (2011) to the 88-item questionnaire by Schneider et al. (2010). Recall periods were typically short and well defined. Potential sources of bias were acknowledged in four publications (Budke et al, 2008, Favrot et al, 2010, Schneider et al, 2010, Iliopoulou et al, 2013). Based on the results of the Scopus search, while some of the validated instruments had been used by different groups of researchers, most had only appeared in peer-reviewed publications by the authors of the instrument.

The types of validation performed for each instrument are summarised in Table 6. There was 87% agreement between scorers NH and ZB after initial scoring of all manuscripts. Lack of agreement was typically due to inadequately described methodology. While many of the instruments had shown some evidence of validity, evidence of assessment of reliability and consistency was infrequent, and no instrument had been validated across all measures. Reporting of the methodology of validation was often incomplete, especially concerning the hypotheses used to test construct and criterion validity. Without a clearly stated hypothesis, the validity of test results was unclear. The format of the instrument designed by Budke et al. (2008) meant inter-rater reliability was not applicable.

The quality of the instruments was also assessed (Table 4). Three publications defined keywords, while a further five discussed existing definitions or domains that should be assessed without stating their own definition. Few instruments had been designed with the constructive input of dog owners, either in the question design or pilot phases. The instrument designed by Budke et al. (2008) was the only one that allowed dog owners to choose and weight the domains that they perceived to be relevant to their dog. Four of the 11 instruments included global QoL ratings.

## Discussion

This rapid review appraised canine QoL instruments available in peer-reviewed, published literature. While many instruments were identified, the majority were novel and unvalidated. The use of multiple novel instruments makes the comparison of outcomes or the assimilation of evidence extremely challenging. We hope that by highlighting validated, high quality QoL instruments, and the areas where they can be applied, more researchers and veterinarians will be encouraged to use relevant existing instruments rather than create novel ones for the same purpose. In common with recent systematic reviews of veterinary literature (Potterton et al, 2012, Downes et al, 2013), detailed methodology was frequently lacking in the papers we included, especially concerning the items included in an instrument. Definitions of QoL or related terms were also rare, confirming the findings of previous reviews (McMillan, 2000, Hewson et al, 2007).

Reporting of methodology and basic data were frequently incomplete in the publications reviewed. Authors of several excluded publications made broad statements in their abstracts about the benefit to QoL of a specific technique or medication without providing any evidence for that assertion. It was necessary to contact the authors of 34 publications to obtain the instrument they used; finding valid contact details for these authors was challenging. Several of the publications reporting validated instruments provided poor details of methodology by which the validation was performed, and in a couple of cases the instrument described in the publication did not entirely match that provided by the author. Reporting guidelines (Gallo et al, 2011, Grindlay et al, 2014) should be used to ensure sufficient information is included in publications to allow for replication. The use of supplementary online material can ensure that, even when tight word limits are imposed, full details of methodology and the complete instrument can be made available.

All 151 publications assessed at the whole paper level included the terms QoL or wellbeing in their abstracts, but few clearly defined these terms, especially in publications containing unvalidated instruments. Concise definitions give readers clarity on whether the construct defined is actually being measured. In many publications, physical health assessment was interpreted as a measure of global QoL; QoL and HRQoL were used interchangeably by many authors. This is not a problem unique to veterinary medicine (Gill, Feinstein, 1994, Smith et al, 1999), but is compounded by the lack of consensus regarding what QoL and HRQoL mean in veterinary medicine. Definitions of QoL have been proposed (McMillan, 2000, Wojciechowska, Hewson, 2005, Wiseman-Orr et al, 2006) but not widely adopted. Some definitions are published in journals that might not be widely read by practicing veterinarians, and others, while extremely clear, are too long to be of practical use. Clear, concise and relevant definitions of HRQoL and QoL in veterinary medicine are needed to address this.

Only 11/52 instruments reviewed in this study had undergone any form of validation, and those instruments had rarely been subsequently used by other groups of researchers. The degree of validation varied greatly between instruments. It is possible that inter-rater reliability was not assessed in any instrument due to the subjective nature of QoL or because many instruments required knowledge of the dog's behaviour over at least 7 days. This should not be a barrier, as consensus is highly relevant when considering dogs in multi-person households. Similarly, criterion validity was rarely assessed, perhaps due to the lack of a reference standard relevant to overall QoL. Validation is an iterative process, and several of the instruments reviewed (e.g. Lynch et al., 2010) were in the earliest stages of this. The reasons for the infrequent use of validated instruments are likely to be multifactorial. Validated instruments have yet to be developed for most areas of canine medicine and surgery. Several validated instruments found in this review were difficult to access, and awareness of their existence is likely to be poor. It is hoped that this review will provide a good synthesis of the available instruments for veterinarians new to this field. Additionally, our study draws attention to the fact that both reliability and validity can, and should, be assessed for subjective constructs such as QoL.

For two instruments reviewed, there were aspects of validation that were not applicable due to the aim or design of the QoL instrument. The aim of the instrument designed by Yeates et al. (2011) was to promote discussion between clients and veterinarians about aspects QoL, and as such, many of the traditional assessments of validity were not relevant. The personalised nature of the instrument designed by Budke et al. (2008) limits the applicability of inter-rater reliability testing. These instruments are no less valuable for this, and it is important to take into account the purpose of the instrument when appraising the quality of its validation.

This rapid review critically appraised the quality of QoL instruments for use in dogs, a practice that has been performed on human QoL instruments for over 20 years (Gill and Feinstein, 1994). One area of quality that is consistently highlighted as important in human healthcare is that patients have a significant role in instrument design (Gill, Feinstein, 1994, Guyatt et al, 1997, Locker, Allen, 2007). The majority of instruments reviewed did not involve dog owners in the design and pilot stages, a concern also highlighted by Yeates and Main (2009).

In evaluating QoL instruments used in human healthcare, Gill and Feinstein (1994) advocated the inclusion of a total QoL score, both to provide a simplified result for clinicians and to promote weighting of different domains of QoL. Three of the validated instruments reviewed here include a total score, but domains of QoL were not weighted differently. The importance of different domains of QoL is likely to differ between dogs. Since dogs comprise a diverse species, individual needs and motivations can also be diverse (King et al, 2012). Furthermore, needs and motivations might be age-dependent, as is the case for humans (Steptoe et al., 2014).

Generic assessments that do not take into account individual differences might not provide an accurate reflection of QoL for any one individual. The inclusion of a global QoL rating item in instruments is common in human healthcare and is viewed as complementary to more structured questions (Gill and Feinstein, 1994). Global ratings allow for the possibility that more structured items might not be suited to every individual, although Yeates and Main (2009) offer an alternative perspective on this point. Another method of reflecting individual differences is to allow the proxy rater to select and weight the domains of QoL to be assessed, based on what they perceive are the needs and interests of each individual assessed. This method was used in the instrument designed by Budke et al. (2008). Such instruments might be less useful for population-level comparisons (e.g. as outcome measures), but could be ideal for assessing changes over time and assisting decision-making in individual patients.

Our rapid review found three different applications for validated QoL instruments: (1) QoL assessment in a disease-specific population (Freeman et al., 2005); (2) as an aid to promoting QoL discussions in veterinary practice (Yeates et al., 2011), and (3) as a generic assessment of QoL (Schneider et al., 2010). Eight of the 11 validated instruments included in the review were designed for the first application. Due to their specificity, those instruments are unlikely to be adopted in general practice, since many dogs present with multiple comorbidities (Robinson et al., 2015). In such a setting, a robust instrument that could be used to discuss and assess QoL in all dogs would maximise its adoption in veterinary practice. The instrument for this purpose is likely to be a form of framework as described by Yeates and Main (2009), which can simply capture what are thought to be the most important, positive constituents of QoL for individual dogs, as described by their caregivers. These could then be optimised and assessed over time, promoting interventions and decisions that truly reflect each dog's QoL. A validated instrument of that design was not found during this review.

This rapid review had a number of limitations. CAB Abstracts and PubMed were used for our search as they have been found to produce the most results when looking for veterinary literature (Grindlay et al., 2012). More evidence might exist in ‘grey literature’ or in additional databases that were not searched due to the time constraints of a rapid review. Many of the authors contacted for further information on a partially reported tool did not reply; letters or reminder emails could have been sent to these authors, but this was outside the scope of a rapid review. One publication included in our rapid review (Hamilton et al., 2012) stated that its intent was not to measure QoL, but since their instrument fitted our inclusion criteria, it was appraised. Instrument development is an iterative process, as reflected in Table 5. Some instruments reviewed were in the early stages of development, and might undergo further refinement in the future. Finally, our review only covered validated instruments for dogs that met the inclusion criteria. Since this is a rapidly moving field, it is likely that new instruments have been published since the review was undertaken.

## Conclusions

Appropriate, validated instruments should be used to assess canine QoL and the use of novel, unvalidated instruments should be discouraged. It is hoped that this review, both by highlighting the validated instruments and by providing checklists for validation and quality, will increase awareness of validated instruments and improve the quality of those used in the future. The majority of validated instruments are suitable for use in dogs with a single disease. This does not reflect the reality of general practice, where many dogs have multiple conditions that need to be considered in an assessment of QoL. However, it is unlikely that a veterinarian in general practice would use multiple instruments. If the assessment of QoL using validated instruments really is to be a ‘central part’ of everyday veterinary practice, further research is required in order to design and validate high quality instruments that are truly fit for this purpose.

## Conflict of interest statement

None of the authors has any financial or personal relationships that could inappropriately influence or bias the content of the paper.

## Figures and Tables

**Table 1:** Summary of information extracted from the publications reviewed.

Information extracted	Potential answers
Was the instrument designed for use in dogs with a specific disease type?	Oncology; gastroenterology; hepatology; cardiorespiratory; dermatology Neurology; orthopaedic; soft tissue; general screening; no
Was the keyword defined?	Free text
What was length of the recall period?	At the time of instrument administration; time (in units given in publication or description of information given); not stated
Was the instrument reproduced, described or referenced?	Free text
Was there any evidence that the authors had attempted to validate the instrument prior to its use to collect clinical data?	Yes/no
Brief description of the instrument in the format available for review^a^	Free text
What was the function of the instrument as stated by the authors?^a^	Free text
Which publications cite this instrument as found by the Scopus search?^a^	Free text
Information relevant to reliability, validity and quality^a^	Table 2, Table 3, Table 4
Who is completing the questions in the instrument?^b^, ^c^	One or more of: owner; veterinarian; clinical investigator; unclear
Was the method behind construction of the instrument described?^b^	Yes; yes some questions adapted from elsewhere; yes, references an unvalidated instrument not found elsewhere in this search; no
Was a scoring or weighting applied to the results?^b^	Yes (explanation of method used); no

a Validated instruments only.

b Unvalidated instruments only.

c All validated instruments were for completion by owners.

**Table 2:** Assessment criteria for reliability (adapted from Taylor and Mills, 2006).

Test	Aim of test	Criteria	Legend for Table 6
Intra-rater reliability	To assess reliability in scoring when one person repeat-scores the animal	Does the same person repeatedly score the same animal under the same conditions within a short time period?	1
Is that time period clearly stated?	2
Is the gap between repeat scores a minimum of 1 week, ideally a minimum of 2 weeks?	3
Is the consistency of scoring between first and subsequent assessments compared? (Tests for comparison are typically correlation coefficients such as the intra-class correlation coefficient, Kappa coefficient, Pearson's, Spearman's Rank or Kendall's tau-b.)	4
Have reliability statistics been assessed against a stated threshold?	5
Inter-rater reliability	To assess reliability in scoring when scorers simultaneously score the same animal	Do multiple people simultaneously score the same animal?	6
Does the methodology describe a circumstance which ensures that the scores of each rater are independent and unbiased by each other?	7
Is the consistency of scores between raters compared? (Tests for comparison are typically correlation coefficients such as the intra-class correlation coefficient, Kappa coefficient, Pearson's, Spearman's Rank or Kendall's tau-b.)	8
Have reliability statistics been assessed against a stated threshold?	9
Test–retest reliability	To assess consistency in scoring when a long period of time has elapsed	Does the same person score the same animal under the same conditions after a considerable time interval? (Length of interval might be constrained by the health condition; criterion 14)	10
Is the time period clearly stated?	11
Is the gap between repeat scores a minimum of 2 weeks? (Longer time periods were preferred.)	12
Is the consistency of scores compared? (Tests for comparison are typically correlation coefficients based upon rank order consistency such as the intra-class correlation coefficient, Spearman's Rank or Kendall's tau-b.)	13
Where relevant, is it acknowledged that for rapidly changing health conditions, this assessment is not always possible, or that time intervals might need to be shorter?	14
Have reliability statistics been assessed against a stated threshold?	15
Internal consistency	To assess whether, if questions are grouped in any form, there is a correlation between questions within the groups	Has an attempt been made to determine whether correlations exist between questions which are grouped together?	16
Is the method of grouping the questions stated?	17
Is the method of grouping appropriate? (Methods include factor analysis and principal component analysis; each has their own criteria for appropriate use.)	18
Has an analysis been performed to look for correlations between questions within groups, factors or components?	19
Is the method of analysis appropriate? (Methods of analysing within group correlations include Cronbach's Alpha and intra-class correlation coefficients.)	20

**Table 3:** Assessment criteria for validity (adapted from Taylor and Mills, 2006).

Test	Aim of test	Criteria	Legend for Table 6
Content validity (face validity is a form of this)	To assess whether individual questions really ask what they are meant to be asking	Has an attempt been made to ensure that the questions in the instrument truly ask what they should? (e.g. do questions in the area of comfort truly ask about comfort?)	21
Is the method by which this has been performed described?	22
Is the method appropriate? (Methods include consultation with a panel of experts which in this context might be veterinarians, dog owners, canine behaviour experts etc.)	23
Construct validity	Whether questions, or groups of questions, ask what they are meant to be asking. This is assessed by comparing constructs which are hypothesised to be related. A construct is something which cannot be proved or objectively measured, e.g. quality of life, happiness.	Has an attempt been made to statistically check whether questions truly assess the broad area which they were designed to assess by comparing the relationships between questions/groups, or between questions/groups and other observable responses? (e.g. questions about comfort should be negatively associated with questions about pain level; and comfort scores should be negatively associated with sleep quality, while pain scores should be positively associated with reduced movement)	24
Have hypotheses about expected positive (convergent) and/or negative (divergent) associations between tested measures been clearly stated before analysis? This is critical to the assessment of construct validity.	25
Is the method by which the assessment has been made described?	26
Is this method appropriate? (Potential methods are numerous but include comparing the distribution of scores to other observable measures, or comparisons between scores within the instrument.)	27
Criterion (concurrent) validity	How this instrument compares to an independent reference standard measure. A criterion is something which can be objectively and definitively measured, e.g. age, a hip score. A measurement of a construct should not be used as the comparator in criterion testing.	Has the instrument been compared to a different instrument/measurement (criterion measure, standard reference test, reference standard) which measures the same thing?	28
Do the authors state that the criterion method used has been validated, or provide a reference?	29
Have hypotheses about expected associations between the instrument and the comparison measure been clearly stated, including the directionality of the expected correlation, before being tested? This is critical to the assessment of criterion validity.	30
Has the time when the criterion measurement was performed been clearly stated (typically at the same time as concurrent validity)?	31
Did the instrument produce results comparable to a reference standard?	32
Criterion (known groups) validity	Whether the instrument can distinguish between groups of veterinary patients, e.g. dogs with different severities of heart disease, or dogs given placebo vs. treatment	Has the instrument been assessed for its ability to distinguish clinically relevant differences between known groups?	33
Have hypotheses about expected associations between the instrument and the comparison measure been clearly stated, including the directionality of the expected correlation, before being tested? This is critical to the assessment of criterion validity.	34
Is the time when the assessment of the known group was performed clearly stated?	35
Has the instrument been shown to distinguish between different populations or groups?	36

**Table 4:** Assessment criteria for, and results of, the quality appraisal of the 11 validated instruments (based on questions by Gill, Feinstein, 1994, Guyatt et al, 1997, Locker, Allen, 2007).

	Are keywords (e.g. QoL) defined within the instrument?	Are the domains of QoL to be measured stated in the publication or instrument?	Do the investigators state why they used this instrument rather than any other?	During a pilot, were owners asked to suggest additional questions which could be included?	Were the questions informed by discussion or qualitative interviews with those who will complete the instrument?	If the authors aimed to measure QoL rather than HRQoL, is the instrument doing so?	Is a single-question overall QoL rating included?	Are multiple items aggregated into a single score?	Are owners asked to indicate which items were personally important to them?	If so is this incorporated into a weighted score?
Brown et al., 2007	N	Y	Y	Y	NS	Y	Y	N	N	NA
Budke et al., 2008	N	NA	Y	NA	NA	Y	Y	NA	Y	Y
Favrot et al., 2010	D	N	N	N	NS	NA	N	N	N	NA
Freeman et al., 2005	N	N	Y	N	N	NA	N	Y	N	NA
Iliopoulou et al., 2013	D	N	Y	N	N	Y	Y	N	N	NA
Lynch et al., 2010	Y	Y	Y	N	Y	Y	Y	N	N	NA
Mullan et al., 2007	Y	Y	Y	N	N	Y	N	N	Y	N
Noli et al., 2011a	Y	N	Y	Y	NS	N	N	Y	N	NA
Schneider et al., 2010	D	Y	Y	N	N	Y	N	N	N	NA
Yazbek et al., 2005	D	Y	N	NS	NS	Y	N	Y	N	NA
Yeates et al., 2011	D	Y	Y	Y	NS	Y	N	N	N	NA

QOL, Quality of life; HRQoL, Health related quality of life; N, no; Y, yes; NA, not applicable; NS, not stated; D, the authors discuss definitions but do not clearly state which definition they have used.

**Table 5:** Summary of information extracted from the 11 validated instruments.

Publication (name of instrument if stated)	Function of instrument as stated by the authors	Brief description of instrument in the format available for review	Was the instrument validated in dogs with a specific disease type?	What was the recall period?	Was the instrument reproduced, described or referenced?	Publications which cite this publication found in Scopus search
Brown et al., 2007; Canine Brief Pain Inventory	Owners' perceptions of the severity and impact of chronic pain on their dogs with osteoarthritis	Two page, 11 question instrument. Four questions on pain, six on function (both numeric scales) and one scale for QoL (Likert-type).	Chronic pain	Previous 7 days	No. Later publications refer to website for download	Brown et al, 2008, Brown et al, 2009, Brown et al, 2013a, Brown et al, 2013b Gordon-Evans et al., 2013 Imhoff et al., 2011 Malek et al., 2012 Sullivan et al., 2013 Walton et al., 2013 Wernham et al., 2011
Budke et al., 2008	Owner-perceived, weighted quality of life assessments for dogs with spinal cord injuries	Owners asked to choose five areas of life/life activity important to their dog, then to weight these using a laminated disc. Separate visual analogue scales for QoL and owner ability to cope with spinal cord injury.	Spinal cord disease	At the time of completion	Adequately described	Levine et al., 2008
Favrot et al., 2010	Impact of atopic dermatitis on health-related quality of life of affected dogs and their owners	One page, 14 question instrument proposed for future use. Thirteen questions regarding QoL in the dog related to its skin disease and one about the QoL of the owners. Likert-type scale.	Skin disease	Since last visit to veterinarian	Reproduced	Linek and Favrot, 2010
Freeman et al., 2005 Functional Evaluation of Cardiac Health	Health-related quality of life in dogs with cardiac disease	Two page, 18 question instrument. All questions relate to how the dog's heart disease has impacted on its comfort or sociability in the preceding 7 days. Likert-type scale.	Cardiac disease	Previous 7 days	Adequately described and available from author	Atkinson et al., 2009 Cunningham et al., 2013 Peddle et al., 2012 Marcondes-Santos et al., 2007 Rutherford et al., 2012
Iliopoulou et al., 2013	Quality of life survey for use in a canine cancer chemotherapy setting	Four page, 30 question instrument. Three sections: 14 questions about how the dog was 6 months previously; 13 questions about the dog's QoL now; three questions about how the owners coped during the chemotherapy. Mixed scale types.	Cancer treated by chemotherapy	At the time of completion and 6 months previously	No. Available from the author	None
Lynch et al., 2010	Health-related quality of life in canine and feline cancer patients	One page, 24 question instrument. Eight sections, each with three questions. Sections on happiness, mental status, pain, appetite, hygiene, hydration, mobility and general health. Likert-type scales apart from the final global QoL question which is a visual analogue scale.	Cancer	At the time of completion	Reproduced	Chon et al., 2012
Mullan et al., 2007	To raise awareness of welfare considerations of pet dogs visiting a veterinary practice	Four page, 39 question instrument. Seven sections: three questions on comfort; three on exercise; three on diet; three on mental stimulation; four on companionship; 16 across two sections on behaviour. Mix of Likert-type and visual analogue scales and one open question.	No	At the time of completion and ‘at their best’	Adequately described and available from author	None
Noli et al., 2011a	Quality of life of dogs with skin diseases and their owners	One page, 15 question instrument. No subdivision into sections, all disease related. Likert-type scale.	Skin disease	Previous seven days	Reproduced	Noli et al., 2011b
Schneider et al., 2010	Multidimensional assessments regarding QoL and the human–animal bond of companion dogs	Four page, 88 question instrument. Four sections: physical (27 questions), psychological (30 questions), social (15 questions) and environment (16 questions). All Likert-type scale.	No	At the time of completion	No. Available from the author^a^	None
Yazbek et al., 2005	Health-related quality-of-life scale for dogs with pain secondary to cancer	One page, 12 question instrument. No subdivision into sections, Likert-type scale.	Cancer	At the time of completion	Reproduced	Flor et al., 2013
Yeates et al., 2011	A participatory tool in order to encourage discussions and decisions about dogs' quality of life	One page, five question instrument. Questions asking owners how well they provide for five ‘needs’. Visual analogue scale.	No	At the time of completion	Adequately described^b^	None

a The instrument provided by the author contains 88 questions; the publication describes a 91 question instrument.

b The illustration of the instrument provided in the publication is different to its description.

**Table 6:** Results of reliability and validity assessment performed on all publications that cited the instrument.^a^

	Reliability and consistency																				Validity															
Intra-rater					Inter-rater				Test–retest						Internal					Content			Construct				Criterion					Known group			
Criteria	1	2	3	4	5	6	7	8	9	10	11	12	13	14	15	16	17	18	19	20	21	22	23	24	25	26	27	28	29	30	31	32	33	34	35	36
Brown et al., 2007	A	A	A	A	A	A	A	A	A	P	P	A	P	P	P	P	P	P	P	P	P	P	P	P	P	P	P	P	P	P	P	P	P	P	P	P
Budke et al., 2008	A	A	A	A	A	N	N	N	N	A	A	A	A	A	A	A	A	A	A	A	P	P	P	P	A	P	P	P	P	A	P	P	P	A	P	P
Favrot et al, 2010	A	A	A	A	A	A	A	A	A	A	A	A	A	A	A	A	A	A	A	A	P	P	P	P	A	P	P	A	A	A	A	A	A	A	A	A
Freeman et al., 2005^b^	A	A	A	A	A	A	A	A	A	P	A	A	P	A	P	P	A	A	A	P	P	P	P	P	A	P	P	A	A	A	A	A	P	P	P	P
Iliopoulou et al., 2013^c^	A	A	A	A	A	A	A	A	A	A	A	A	A	A	A	A	A	A	A	A	A	A	A	P	A	P	P	A	A	A	A	A	P	A	P	P
Lynch et al., 2010	A	A	A	A	A	A	A	A	A	A	A	A	A	A	A	A	A	A	A	A	P	P	P	A	A	A	A	A	A	A	A	A	A	A	A	A
Mullan et al., 2007	P	P	A	P	A	A	A	A	A	A	A	A	A	P	A	A	A	A	A	A	P	P	P	P	P	P	P	A	A	A	A	A	A	A	A	A
Noli et al., 2011a^d^	A	A	A	A	A	A	A	A	A	A	A	A	A	A	A	P	P	P	P	P	P	P	P	P	A	P	P	A	A	A	A	A	P	P	P	P
Schneider et al., 2010	A	A	A	A	A	A	A	A	A	A	A	A	A	A	A	P	P	P	P	P	A	A	A	P	A	P	P	A	A	A	A	A	P	P	P	P
Yazbek et al., 2005	A	A	A	A	A	A	A	A	A	A	A	A	A	A	A	A	A	A	A	A	A	A	A	A	A	A	A	A	A	A	A	A	P	A	P	P
Yeates et al., 2011^e^	A	A	A	A	A	A	A	A	A	A	A	A	A	A	A	A	A	A	A	A	P	P	P	A	A	A	A	A	A	A	A	A	A	A	A	A

A, absent in any of the publications assessed which contain this instrument; P, present in any of the publications assessed which contain this instrument; N, not applicable for this instrument.

a Assessment criteria in Table 2, Table 3.

b Heart failure as assessed by the International Small Animal Cardiac Health (ISACH) score was considered to be a construct, not a criterion measure, therefore criterion validity was not performed.

c Criterion 27 was likely to have been achieved, but the description of the methodology was inadequate to allow for replication.

d The criterion measures used did not fulfil the accepted definitions used in this review.

e The context in which this instrument was used means that construct and criterion validity were not relevant, but they could in theory be assessed.

## Appendix Supplementary material

Table S1 Instruments used for the assessment of quality of life in dogs without a description of their prior validation

## Footnotes

1 See: WHOQOL-BREF: Introduction, administration, scoring and generic version of the assessment. http://www.who.int/mental_health/media/en/76.pdf (accessed 10 December 2014).

2 See: Choi, B.C.K., Pak, A.W.P., 2005. A catalog of biases in questionnaires. In: Preventing Chronic Disease (Serial online), http://www.cdc.gov/pcd/issues/2005/jan/04_0050.htm, pp. 1–13 (accessed 7 December 2014).

3 See: VetSRev Database of systematic reviews; http://webapps.nottingham.ac.uk/refbase/ (accessed 10 November 2014).

4 See: Oxford English Dictionary, 3rd edition, http://www.oed.com/ (accessed 2 March 2014).
