# Supplementary material for: Quality of life assessment in domestic dogs: An evidence-based rapid review
Source: Vet J. 2015 Nov;206(2):203–12. doi: 10.1016/j.tvjl.2015.07.016 (PMC4641869; doi:10.1016/j.tvjl.2015.07.016)
Supplement: Table S1 — Instruments used for the assessment of quality of life in dogs without a description of their prior validation [file mmc1.docx]

**Supplementary Data – Table 1**

| Reference | Was the instrument designed for use in dogs with a specific disease type? | Are the keywords defined? | Was the instrument reproduced, adequately described or referenced in the publication? | Who is completing the questions in the instrument? | Is the method behind construction of the instrument described? | What was the recall period? | Is a scoring or weighting applied to the results? |
| --- | --- | --- | --- | --- | --- | --- | --- |
| [Ahlstrom et al., 2010](#_ENREF_1) | Dermatology | No | Yes | Owner | No | No recall needed | Yes, score |
| [Amberger et al., 2004](#_ENREF_2) | Cardiology | No | Yes | Clinical investigator (veterinarian) | No | Three months | No |
| [Bauer et al., 1992](#_ENREF_3) | Neurology | No | Yes | Owner | Yes in that some questions adapted from elsewhere | Since before paralysis (minimum of 3 months, median 20 months | Yes, score |
| [Bellei et al., 2011](#_ENREF_4) | Neurology | No | Yes | Unclear but in hospital so vet or technician | Yes in that some questions were adapted from elsewhere | No recall needed | Yes, score |
| [Boothe et al., 1996](#_ENREF_5) | Hepatology | No | Yes | Vet and owner | No | Since diagnosis (median 24 months) | No |
| [Bowles et al., 2010](#_ENREF_6) | Oncology | No | Yes | Owner | No | Asks owners to rate QoL before cancer, after diagnosis but before treatment and then at its best and worst during treatment; mean 22.7 months | No |
| [Brissot et al., 2004](#_ENREF_7) | Soft tissue surgery | No | Yes | Owner | No | Median 26.6 months | No |
| [Bronden et al., 2003](#_ENREF_8) | Oncology | No | No | Owner | No | Since chemotherapy, unclear how long | No |
| [Bulmer et al., 2006](#_ENREF_9) | Cardiology | No | Yes | Owner | Yes in that some questions were adapted from elsewhere | No recall needed | No |
| [Chretin et al., 2007](#_ENREF_10) | Oncology | No | Yes | Unclear from methods. Likely by vet. | No | No recall needed | No |
| [Cook et al., 2008](#_ENREF_11) | Orthopaedics | No | Yes | Owner | Yes | Since before surgery, no clear mean available | No |
| [Craven et al., 2004](#_ENREF_12) | Gastroenterology | Yes | Yes | Owner | No | At diagnosis (median 9.5 months), and at the time of tool administration | No |
| [Crawford et al., 2012](#_ENREF_13) | Oncology | No | Yes | Owner | No | Up to a minimum of 6 years previously based on inclusion criteria. | No |
| [Davies 2012](#_ENREF_14) | General screening | No | Yes | Owner | No | Not time framed | No |
| [Denneberg and Egenvall, 2009](#_ENREF_15) | Oncology | No | Yes | Owner | No | Recalls time of treatment, less than 2 years | No |
| [Dewey et al., 2007](#_ENREF_16) | Neurology | No | Yes | Owner | No | Since surgery, mean 11.2 months | No |
| [Fox et al., 1997](#_ENREF_17) | Oncology | No | Yes | Owner | No | Since before surgery, mean of 44 months | No |
| [Gemmill et al., 2012](#_ENREF_18) | Orthopaedics | No | No | Owner | Yes, uses an unvalidated tool not found elsewhere in search | Minimum of 6 months | No |
| [Hamilton et al., 2012](#_ENREF_19) | Oncology | No | Yes | Owner | No | Not stated. Questionnaire administered before each treatment, so presumably since last visit but not explicit. | No |
| Hielm-Bjorkman et al., 2009 | Orthopaedics | No | Yes | Owner | No | Since the start of the study | Yes, score |
| [Holt and Durdey, 1999](#_ENREF_21) | Oncology | No | Yes | Owner | No | Since before surgery, at least up to 4 years in some cases | No |
| [Inagawa et al., 2005](#_ENREF_22) | Neurology | No | Yes | Owner | No | At the time of tool administration | Yes, score |
| [Kishi et al., 2013](#_ENREF_23) | Orthopaedics | No | Yes | Owner | Yes in that some questions were adapted from elsewhere | Mean 27.5 months | No |
| [Laiju Philip et al., 2010](#_ENREF_24) | Anaesthesia | No | Yes | Clinical investigator | Yes, uses two unvalidated tools not found elsewhere in search | No recall needed | No |
| [London et al., 2009](#_ENREF_25) | Oncology | No | Yes | Owner | No | Not stated | Yes, score |
| [Lord and Podell, 1999](#_ENREF_26) | Neurology | No | Yes | Owner | Yes in that some questions were adapted from elsewhere | Since before treatment (range 6 months-2 years) | Yes, score |
| [Mallery et al., 1999](#_ENREF_27) | Cardiology | No | No | Owner | No | Not defined | No |
| [McMillan et al., 2012](#_ENREF_28) | Oncology | No | No | Owner | No | Median 15 months | Scored by vets in response to questions |
| [Mellanby et al., 2003](#_ENREF_29) | Oncology | No | Yes | Owner | No | Published in 2003, owners contacted with dogs which were treated from 1994 to 2001 so potentially 9 years | No |
| [Milner 2006](#_ENREF_30); Milner et al., 2006 | Gastroenterology | No | No | Owner | No | Median 18 months | No |
| [O'Grady et al., 2008](#_ENREF_31) | Cardiology | No | Yes | Owner | No | Not stated | Yes, score |
| [Oyama et al., 2007](#_ENREF_33) | Cardiology | No | No | Owner | Yes in that some questions were adapted from elsewhere | No recall needed | No |
| [Oyama et al., 2008](#_ENREF_32) | Cardiology | No | No | Owner | No | No recall needed | No |
| [Plessas et al., 2012](#_ENREF_34) | Neurology | No | No | Owner | No | Not stated | Yes, scored by vet on basis of owner responses |
| [Rau et al., 2010](#_ENREF_35) | Oncology | No | Yes | Owner | Yes in that some questions were adapted from elsewhere | No recall needed | No |
| [Rivera et al., 2013](#_ENREF_36) | Oncology | No | Yes | Vet | Yes in that some questions were adapted from elsewhere | No recall needed | No |
| [Roberts et al., 1987](#_ENREF_37) | Oncology | No | Yes | Vet | No | No recall needed | No |
| [Rutz et al., 2004](#_ENREF_38) | Gastroenterology | No | Yes | Owner | No | No recall needed | No |
| [Trostel and Frankel, 2010](#_ENREF_39) | Soft tissue surgery | No | Yes | Owner | No | Up to 28.5 months | No |
| [Watson and Herrtage, 1998](#_ENREF_40) | Hepatology | No | Yes | Owner | No | Since before treatment (mean for those alive 36 months) | No |
| [Westermarck et al., 1990](#_ENREF_41) | Gastroenterology | No | Yes | Owner | No | No recall needed | Yes, score |

**References for publications listed in electronic material**

Ahlstrom, L.A., Mason, K.V., Mills, P.C., 2010. Barazone decreases skin lesions and pruritus and increases quality of life in dogs with atopic dermatitis: a randomized, blinded, placebo-controlled trial. Journal of Veterinary Pharmacology and Therapeutics 33, 573-582.

Amberger, C., Chetboul, V., Bomassi, E., Rougier, S., Woehrle, F., Thoulon, F., group, F., 2004. Comparison of the effects of imidapril and enalapril in a prospective, multicentric randomized trial in dogs with naturally acquired heart failure. Journal of Veterinary Cardiology 6, 9-16.

Bauer, M., Glickman, N., Glickman, L., Toombs, J., Golden, S., Skowronek, C., 1992. Follow-up study of owner attitudes toward home care of paraplegic dogs. Journal of the American Veterinary Medical Association 200, 1809-1816.

Bellei, E., Roncada, P., Pisoni, L., Joechler, M., Zaghini, A., 2011. The use of fentanyl-patch in dogs undergoing spinal surgery: plasma concentration and analgesic efficacy. Journal of Veterinary Pharmacology and Therapeutics 34, 437-441.

Boothe, H.W., Howe, L.M., Edwards, J.F., Slater, M.R., 1996. Multiple extrahepatic portosystemic shunts in dogs: 30 cases (1981-1993). Journal of the American Veterinary Medical Association 208, 1849-1854.

Bowles, D.B., Robson, M.C., Galloway, P.E., Walker, L., 2010. Owner's perception of carboplatin in conjunction with other palliative treatments for cancer therapy. Journal of Small Animal Practice 51, 104-112.

Brissot, H.N., Dupre, G.P., Bouvy, B.M., 2004. Use of laparotomy in a staged approach for resolution of bilateral or complicated perineal hernia in 41 dogs. Veterinary Surgery 33, 412-421.

Bronden, L.B., Rutteman, G.R., Flagstad, A., Teske, E., 2003. Study of dog and cat owners' perceptions of medical treatment for cancer. Veterinary Record 152, 77-80.

Bulmer, B.J., Sisson, D.D., Oyama, M.A., Solter, P.F., Grimm, K.A., Lamont, L., 2006. Physiologic VDD versus nonphysiologic VVI pacing in canine 3rd-degree atrioventricular block. Journal of Veterinary Internal Medicine 20, 257-271.

Chretin, J.D., Rassnick, K.M., Shaw, N.A., Hahn, K.A., Ogilvie, G.K., Kristal, O., Northrup, N.C., Moore, A.S., 2007. Prophylactic trimethoprim-sulfadiazine during chemotherapy in dogs with lymphoma and osteosarcoma: a double-blind, placebo-controlled study. Journal of Veterinary Internal Medicine 21, 141-148.

Cook, J.L., Hudson, C.C., Kuroki, K., 2008. Autogenous osteochondral grafting for treatment of stifle osteochondrosis in dogs. Veterinary Surgery 37, 311-321.

Craven, M., Simpson, J.W., Ridyard, A.E., Chandler, M.L., 2004. Canine inflammatory bowel disease: retrospective analysis of diagnosis and outcome in 80 cases (1995-2002). Journal of Small Animal Practice 45, 336-342.

Crawford, A.H., Tivers, M.S., Adamantos, S.E., 2012. Owner assessment of dogs' quality of life following treatment of neoplastic haemoperitoneum. Veterinary Record 170, 566-567.

Davies, M., 2012. Geriatric screening in first opinion practice - results from 45 dogs. Journal of Small Animal Practice 53, 507-513.

Denneberg, N.A., Egenvall, A., 2009. Evaluation of dog owners' perceptions concerning radiation therapy, In: Acta Veterinaria Scandinavica. BioMed Central, <http://www.actavetscand.com/content/51/1/19>, p. 10.

Dewey, C.W., Marino, D.J., Bailey, K.S., Loughin, C.A., Barone, G., Bolognese, P., Milhorat, T.H., Poppe, D.J., 2007. Foramen magnum decompression with cranioplasty for treatment of caudal occipital malformation syndrome in dogs. Veterinary Surgery 36, 406-415.

Fox, L.E., Geoghegan, S.L., Davis, L.H., Hartzel, J.S., Kubilis, P., Gruber, L.A., 1997. Owner satisfaction with partial mandibulectomy or maxillectomy for treatment of oral tumors in 27 dogs. Journal of the American Animal Hospital Association 33, 25-31.

Gemmill, T.J., Pink, J., Clarke, S.P., McKee, W.M., 2012. Total hip replacement for the treatment of atraumatic slipped femoral capital epiphysis in dogs. Journal of Small Animal Practice 53, 453-458.

Hamilton, M.J., Sarcornrattana, O., Illiopoulou, M., Xie, Y., Kitchell, B., 2012. Questionnaire-based assessment of owner concerns and doctor responsiveness: 107 canine chemotherapy patients. Journal of Small Animal Practice 53, 627-633.

Hielm-Bjorkman, A.K., Rita, H., Tulamo, R.M., 2009. Psychometric testing of the Helsinki chronic pain index by completion of a questionnaire in Finnish by owners of dogs with chronic signs of pain caused by osteoarthritis. Am J Vet Res 70, 727-734.

Holt, P.E., Durdey, P., 1999. Transanal endoscopic treatment of benign canine rectal tumours: preliminary results in six cases (1992 to 1996). Journal of Small Animal Practice 40, 423-427.

Inagawa, K., Seki, S., Bannai, M., Takeuchi, Y., Mori, Y., Takahashi, M., 2005. Alleviative effects of gamma-aminobutyric acid (GABA) on behavioral abnormalities in aged dogs. Journal of Veterinary Medical Science 67, 1063-1066.

Kishi, E.N., Hulse, D., Raske, M., Saunders, W.B., Beale, B.S., 2013. Extra-articular stabilization of the canine cranial cruciate ligament injury using Arthrex Corkscrew and FASTak anchors. Open Journal of Veterinary Medicine 3, 156-160.

Laiju Philip, M., Devanand, C.B., Martin, J.K.D., Amma, T.S., 2010. Pre-operative dextrose infusion to diminish post-operative stress and recovery in elective canine ovariohysterectomy - evaluation of a novel approach. Veterinary World 3, 88-89.

London, C.A., Malpas, P.B., Wood-Follis, S.L., Boucher, J.F., Rusk, A.W., Rosenberg, M.P., Henry, C.J., Mitchener, K.L., Klein, M.K., Hintermeister, J.G., et al., 2009. Multi-center, placebo-controlled, double-blind, randomized study of oral toceranib phosphate (SU11654), a receptor tyrosine kinase inhibitor, for the treatment of dogs with recurrent (either local or distant) mast cell tumor following surgical excision. Clinical Cancer Research 15, 3856-3865.

Lord, L.K., Podell, M., 1999. Owner perception of the care of long-term phenobarbital-treated epileptic dogs. Journal of Small Animal Practice 40, 11-15.

Mallery, K.F., Freeman, L.M., Harpster, N.K., Rush, J.E., 1999. Factors contributing to the decision for euthanasia of dogs with congestive heart failure. Journal of the American Veterinary Medical Association 214, 1201-1204.

McMillan, S.K., Knapp, D.W., Ramos-Vara, J.A., Bonney, P.L., Adams, L.G., 2012. Outcome of urethral stent placement for management of urethral obstruction secondary to transitional cell carcinoma in dogs: 19 cases (2007-2010). Journal of the American Veterinary Medical Association 241, 1627-1632.

Mellanby, R.J., Herrtage, M.E., Dobson, J.M., 2003. Owners' assessments of their dog's quality of life during palliative chemotherapy for lymphoma. Journal of Small Animal Practice 44, 100-103.

Milner, H.R., 2006. The role of surgery in the management of canine anal furunculosis. A review of the literature and a retrospective evaluation of treatment by surgical resection in 51 dogs. New Zealand Veterinary Journal 54, 1-9.

O'Grady, M.R., Minors, S.L., O'Sullivan, M.L., Horne, R., 2008. Effect of pimobendan on case fatality rate in Doberman Pinschers with congestive heart failure caused by dilated cardiomyopathy. Journal of Veterinary Internal Medicine 22, 897-904.

Oyama, M.A., Rush, J.E., O'Sullivan, M.L., Williams, R.M., Rozanski, E.A., Petrie, J.P., Sleeper, M.M., Brown, D.C., 2008. Perceptions and priorities of owners of dogs with heart disease regarding quality versus quantity of life for their pets. Journal of the American Veterinary Medical Association 233, 104-108.

Oyama, M.A., Sisson, D.D., Prosek, R., Bulmer, B.J., Luethy, M.W., Fuentes, V.L., 2007. Carvedilol in dogs with dilated cardiomyopathy. Journal of Veterinary Internal Medicine 21, 1272-1279.

Plessas, I.N., Rusbridge, C., Driver, C.J., Chandler, K.E., Craig, A., McGonnell, I.M., Brodbelt, D.C., Volk, H.A., 2012. Long-term outcome of Cavalier King Charles spaniel dogs with clinical signs associated with Chiari-like malformation and syringomyelia, In: Veterinary Record. group.bmj.com, <http://veterinaryrecord.bmj.com/content/171/20/501.full.html>, p. 5.

Rau, S.E., Barber, L.G., Burgess, K.E., 2010. Efficacy of maropitant in the prevention of delayed vomiting associated with administration of doxorubicin to dogs. Journal of Veterinary Internal Medicine 24, 1452-1457.

Rivera, P., Akerlund-Denneberg, N., Bergvall, K., Kessler, M., Rowe, A., Willmann, M., Persson, G., Kastengren Froberg, G., Westberg, S., von Euler, H., 2013. Clinical efficacy and safety of a water-soluble micellar paclitaxel (Paccal Vet) in canine mastocytomas. Journal of Small Animal Practice 54, 20-27.

Roberts, S.M., Lavach, J.D., Severin, G.A., Withrow, S.J., Gillette, E.L., 1987. Ophthalmic complications following megavoltage irradiation of the nasal and paranasal cavities in dogs. Journal of the American Veterinary Medical Association 190, 43-47.

Rutz, G.M., Steiner, J.M., Bauer, J.E., Williams, D.A., 2004. Effects of exchange of dietary medium chain triglycerides for long-chain triglycerides on serum biochemical variables and subjectively assessed well-being of dogs with exocrine pancreatic insufficiency. Am J Vet Res 65, 1293-1302.

Trostel, C.T., Frankel, D.J., 2010. Punch resection alaplasty technique in dogs and cats with stenotic nares: 14 cases. Journal of the American Animal Hospital Association 46, 5-11.

Watson, P.J., Herrtage, M.E., 1998. Medical management of congenital portosystemic shunts in 27 dogs--a retrospective study. Journal of Small Animal Practice 39, 62-68.

Westermarck, E., Wiberg, M., Junttila, J., 1990. Role of feeding in the treatment of dogs with pancreatic degenerative atrophy. Acta Veterinaria Scandinavica 31, 325-331.
